# Supplementary material for: The Effects of Low-Energy Moderate-Carbohydrate (MCD) and Mixed (MixD) Diets on Serum Lipid Profiles and Body Composition in Middle-Aged Men: A Randomized Controlled Parallel-Group Clinical Trial
Source: Int J Environ Res Public Health. 2020 Feb 19;17(4):1332. doi: 10.3390/ijerph17041332 (PMC7068541; doi:10.3390/ijerph17041332)
Supplement: Supplementary file 1 [file ijerph-17-01332-s001.pdf]

Typical one-day menu with recipe in the CD

Breakfast
Eggs with bacon with bread
Eggs 200g, Bacon 60g, Wheat bread 90g, Butter 15g,

Preparation: Fry bacon and add eggs. Fry to cut eggs. Bread spread with butter

Lunch
Sandwiches with ham and chees
Wheat bread 60g, Butter 5g, Turkey ham 20g, Gouda cheese 15g, Tea 250ml, sugar 10g

Preparation: Bread spread with butter, place ham and cheese. Sugar add to tea

Dinner
Frozen dumplings with minced pork
Dumplings 350g , butter 10g

Preparation: Dumplings cook according to the instructions on the package. Heat the butter in a pan then add the cooked dumplings to the butter. Fry about 5 minutes.

Supper
Sandwiches with Parma ham and marinated artichokes
Wheat bread 60g, Parma ham 40g, Fromage cheese 20g, artichokes 50g, Bear 500ml

Preparation: : Bread spread with cheese place ham and artichokes.

Typical one-day menu with recipe in the MCD

Breakfast
Fruit salad with millet
Millet 60g,Banana 70g,Raspberries 125g,Almonds 30g,Coconut oil 15g

Preparation: Boil the porridge according to the instructions on the packaging, then fry in a pre-heated oil pan. then add chopped fruit porridge: banana, raspberries and chopped almonds. Sprinkle with fresh mint leaves and mix.

Lunch
Tuna salad with avocado and radish, bread
Tuna 160g,Lettuce 20g,Radish 15g,Avocado 80g,Olive oil 10g,Lemon juice 20g,Whole grain rye bread 60g

Preparation: Rinse the lettuce, dry, mix with chopped radish, drizzle with a mixture of olive oil and lemon juice. Add chopped avocado and serve with tuna

Dinner
Beef stew with vegetables
Beef stew 250g ,Potatoes 150g,Onion 75g,Garlic 10g,Carrot 50g,Rapeseed oil 20g,Juniper berries 2g,Sage 2g,Bay leaf 2g,Black Pepper 2g,Rosemary 2g,Tomato juice 200g

Preparation: The meat cleaned and cut into cubes, rub pepper, add half of the oil and particulate juniper berries, bay leaf, sage leaf. Marinate overnight. Chopped onions, carrots, cut into slices, diced potatoes, garlic squeeze by crushed. Heat the oil, add onions, garlic and meat, fry. Then add the other vegetables, whole pour tomato juice, add a sprig of rosemary. Bring to the boil and simmer 1.5 - 2 hours, until the meat is soft.

Supper
Herring in tomato sauce with sandwiches
Herring in tomato sauce 150g ,Whole grain rye bread 60g ,Butter 5g,Lettuce 10g,Radish 15g, Cucumber 50g

Preparation: Bread spread with butter. Put the lettuce leaf and cucumber and radish on the bread. Transfer the herring to a plate. Eat with a sandwich.

Typical one-day menu with recipe in the MixD

Breakfast
Quinoa with raspberries and Goji berries
Quinoa 100g, raspberries 100g, Milk 1.5%of fat 150ml, Goji berries 25g

Preparation: Pour quinoa with milk, cook until it absorbs all the liquid (about 15-20 minutes). Add fruit and mix.

Lunch
Sandwiches with ham and tomato, yogurt with pumpkin seeds, fruit
Graham bread 60g, Butter 10g, Lettuce 10g, Turkey ham 60g, Tomato 60g, Natural yogurt 10g, pumpkins seeds 10g, Grapefruit 150g

Preparation: Bread spread with butter, place lettuce, ham and tomato cut into slices. Pumpkin seeds add to the yogurt and mix.

Dinner
Spaghetti with ground beef
Spaghetti pasta 120g, Ground beef 150g, Onion 25g, Garlic 5g, Coconut oil 10g, Oregano 3g, Thyme 3g, Black pepper 3g, tomato puree 50g, Cherry tomatoes 50g

Preparation: Prepare pasta according to the recipe on the packaging. To minced meat add spices (paprika, pepper, oregano and thyme) and mix. In a skillet heat the oil, add the crushed garlic, pressed and after a while the meat. Fry, then add the chopped onion, half a glass of water and tomato paste. Simmer a few minutes. Combine the sauce with prepared pasta, mix gently. Garnish with cherry tomato halves.

Supper
Mackerel paste with bread
Smoked mackerel 100g, Graham bread 60g, Pickled cucumber 50g, Red pepper 100g, Cream cheese 100g, Onion 20g, Chive 5g

Preparation: Remove the skin from the fish fillet and divide into small pieces. Cucumber, onion, pepper and chives cut. Mix all ingredients with cream cheese, season with pepper to taste.Apply pasta on bread.
